# Managing the COVID-19 health crisis: a survey of Swiss hospital pharmacies

**DOI:** 10.1186/s12913-023-10105-6

**Published:** 2023-10-20

**Authors:** Laurence Schumacher, Yassine Dhif, Pascal Bonnabry, Nicolas Widmer

**Affiliations:** 1 Specialised Centre for Emergency and Disaster Pharmacy, Institute of Pharmaceutical Sciences of Western Switzerland, University of Geneva, Geneva, Switzerland; 2 Pharmacy, Geneva University Hospitals, Geneva, Switzerland; 3 Pharmacy of the Eastern Vaud Hospitals, Rennaz, Switzerland

**Keywords:** Disasters, Response, COVID-19, Lessons learned, Pandemics, Health facilities, Pharmacy service, Hospital

## Abstract

**Background:**

The COVID-19 pandemic strained healthcare systems immensely as of 2020. Switzerland’s hospital pharmacies’ responses during the first wave were surveyed with a view to improving the quality of pharmaceutical management in future health crises.

**Methods:**

An online survey was sent to the heads of all of Switzerland’s hospital pharmacies. The questionnaire was organised into eleven sections of questions covering many topics regarding the management of COVID-19’s first wave. Data collection occurred from May to June 2020.

**Results:**

Analyses were performed using the 43 questionnaires (66%), with at least one answer per questionnaire, out of 65 distributed. Seventeen of 41 pharmacies responding (41%) had existing standard operating procedures or pandemic plans and 95% of these (39/41) set up crisis management steering committees. Twenty-nine of 43 pharmacies responding (67%) created new activities to respond to the pandemic’s specific needs. Twenty-six of 39 pharmacies responding (67%) created new drug lists for: COVID-19-specific treatments (85%; 22/26), sedatives (81%; 21/26), anaesthetics (77%; 20/26) and antibiotics (73%; 19/26). Drug availability in designated COVID-19 wards was managed by increasing existing stocks (54%; 22/41 pharmacies) and creating extra storage space (51%; 21/41). Two drugs generated the greatest concern about shortages: propofol (49%; 19/39 pharmacies) and midazolam (44%; 17/39). Remdesivir stocks ran out in 26% of pharmacies (10/39). Twelve of 43 pharmacies (28%) drafted specific new documents to respond to medical needs regarding drug administration, 12 (28%) did so for drug preparation and 10 (23%) did so for treatment choices.

**Conclusions:**

Switzerland’s hospital pharmacies encountered many challenges related to the COVID-19 crisis and had to find solutions quickly, effectively and safely. The survey highlighted the key role that hospital pharmacies played in many aspects of the pandemic by providing logistical and clinical support to medical and nursing care teams. The lessons and experiences outlined could be used to improve the quality of hospital pharmacies’ readiness for similar future events.

**Supplementary Information:**

The online version contains supplementary material available at 10.1186/s12913-023-10105-6.

## Key points

The COVID-19 pandemic generated unprecedented global demand for specific drugs, hand sanitiser solution and other therapeutic products, particularly in critical care settings, highlighting the essential role of hospital pharmacists in such crises.

Key COVID-19 responses at the hospital pharmacy level included staff flexibility regarding changes in roles and procedures, communication, teamwork and solidarity, and the need to prepare business continuity plans and management dashboards.

Managing and coping with a complex pandemic reveals the importance of involving hospital pharmacists in pandemic response steering committees at many levels. Pharmacists’ lived experiences during the pandemic should be described and evaluated by hospital pharmacies worldwide, both to raise awareness and to guide appropriate policy responses when the next crisis occurs.

**Supplementary Information:**

The online version contains supplementary material available at 10.1186/s12913-023-10105-6.

## Background

2020 was marked by the coronavirus disease 2019 (COVID-19) pandemic and will undoubtedly go down in history. In December 2019, the first cases of pneumonia due to a new, previously unknown coronavirus were reported in the city of Wuhan, capital of Hubei Province in China [1]. In February 2020, the World Health Organization (WHO) officially named this new virus SARS-CoV-2 and the disease it caused, COVID-19, short for coronavirus disease 2019. Human-to-human transmission was rapidly confirmed [2].

The first case of COVID-19 in Switzerland was confirmed on 25 February 2020 in the canton of Ticino. On 28 February, in accordance with the Federal Act on Epidemics (EpidA), the Swiss Federal Council declared a ‘special situation’ by presenting its COVID-19 Ordinance and prohibiting gatherings of more than 1,000 people. At the beginning of March 2020, the number of cases detected had become increasingly important across Europe, mainly in Italy, where the European outbreak started [3]. On 11 March, the WHO declared the COVID-19 epidemic to be a pandemic. On 12 March, Switzerland had the third highest prevalence of COVID-19 of any European country affected by the coronavirus. On 16 March, the Swiss Federal Council declared an ‘extraordinary situation’ (Art. 7 EpidA) and updated COVID-19 Ordinance 2 with new measures: the closure of non-essential businesses (including restaurants and leisure facilities) and partial border closures [4]. It also progressively mobilised several conscript units of the Swiss Armed Forces to assist cantons with healthcare, logistics and security [5]. Switzerland’s healthcare system never reached saturation point, despite it being one of the most highly affected countries globally, with prevalence increasing from 7.2 to 357 cases per 100,000 inhabitants between 9 March and 19 May 2020 [6]. In summer 2023, Switzerland had already faced nine waves and had totalled more than 67,000 hospitalisations and 14,000 deaths due to a COVID-19 infection since the beginning of the pandemic.

Hospital pharmacy services were in high demand, especially during the pandemic’s first wave, because they often bore the primary responsibility for supplying hospitals with therapeutic products. However, in disaster contexts, pharmacists are often forgotten or disregarded despite being essential members of disaster healthcare teams. Indeed, the literature revealed little interest in pharmacists’ roles in disasters until the events of 9/11, 2001 [7]. Prior to this, pharmacists’ accepted roles generally involved their well-established contributions to logistics and supply chain management [8]. Pharmacists’ additional contributions to disaster health management in general [9, 10], and to pandemics [11] in particular, were identified following disasters like Hurricane Katrina in 2005 [12] or Australia’s bushfire disaster in 2019 [13].

Many studies published since the beginning of the COVID-19 pandemic have examined the public health response, but few have focused specifically on hospital pharmacy responses or pharmacists’ [14–18]. Some have described hospital pharmacy responses in Europe but with few details [19, 20]. Any publications that report on the details of the pandemic experience could prove essential to improving the quality of preparations for another such event in the future.

The present study therefore aimed to provide an overview of the actions undertaken by hospital pharmacies across Switzerland as they responded to the challenges encountered during the first wave of the COVID-19 pandemic. It also sought to provide important information and new proposals to improve the quality of pharmaceutical management in future health crises.

## Method

We distributed an online questionnaire designed using the SurveyMonkey® platform (SurveyMonkey, San Mateo, CA, USA) to a pre-established list of pharmacy heads in Switzerland’s hospital pharmacies. Many of the survey questions emerged from our own experiences and from initial feedback from fellow hospital pharmacists. The Swiss Association of Public Health Administration and Hospital Pharmacists (GSASA) approved the survey’s distribution in English to facilitate its uniform dissemination across the country’s different linguistic regions.

Questions were organised into eleven sections and covered general information about the respondent, the hospital pharmacy and the hospital, management of this crisis, human resources management, drugs used primarily in intensive care units (ICUs), drugs used specifically for treating SARS-CoV-2, drug management in designated COVID-19 units (including ICUs), hygiene, support for medical and nursing care teams, care management for patients recovering from COVID-19, other problems encountered, and future perspectives. There was a total of 67 questions, and the questionnaire used an adaptive design to create a custom pathway through the survey depending on the respondent’s answers. We used conditional branching to create a custom path through the survey, depending on the respondent’s answer. Thus, not every participant needed to answer every question, and each question, therefore, had a different number of respondents. To manage non-response errors and missing data, each question uses its own denominator. Most of the questions were multiple-choice and permitted multiple answers. All the questions are available in Appendix I.

Data were collected between 19 May and 19 June 2020. The survey was sent out to the professional email address of every GSASA-affiliated head hospital pharmacist (n = 65) in Switzerland. Email reminders were sent two, three and four weeks after the first email. Data were analysed using standard descriptive statistics. Means and proportions were calculated using the total number of participants responding to the particular question. Responses left blank were coded as missing data and handled using a list-wise deletion method. The respondents who only answered questions about general information were excluded.

The Human Research Ethics Committee of the Canton of Geneva waived the need for ethical oversight because the research protocol required no patient data. Raw data were exported into Microsoft Excel® software (version 2013, Microsoft Corporation, Redmond, WA, USA) for our descriptive statistical analysis.

## Results

### General information

We received responses from 43 of the 65 head pharmacists who were sent surveys—a 66% participation rate. The number of answers for each question is available in Appendix II. Survey completion took participants an average of about 39 min. The characteristics of participating pharmacies are shown in Table 1.

**Table 1:** Characteristics of participating pharmacies

Parameters		N (%)
**Linguistic region of Switzerland**	German-speaking	34 (79)
	French-speaking	8 (19)
	Italian-speaking	1 (2)
**Staff working in the hospital pharmacy**	< 50	35 (81)
	50–100	5 (12)
	> 100	3 (7)
**Sites supplied by the hospital pharmacy**	1–5	31 (72)
	6–10	7 (16)
	> 11	5 (12)
**Number of beds in the hospital (total)**	< 300	26 (60)
	300–1000	15 (35)
	> 1000	2 (5)
**Number of beds in the intensive care unit/intermediate care unit**	< 10	22 (51) / 30 (70)
	10–20	15 (35) / 7 (16)
	21–30	3 (7) / 3 (7)
	31–50	3 (7) / 1 (2)
	> 50	0 (0) / 2 (5)
**Number of beds in the internal medicine department**	< 50	2 (5)
	50–100	7 (16)
	101–200	19 (44)
	201–300	5 (12)
	301–500	5 (12)
	> 500	5 (12)

### Managing the crisis

During the first wave of the COVID-19 outbreak, 41% (17/41) of hospital pharmacies were able to refer to previously prepared internal standard operating procedures or plans for disaster management, whether for disasters in general (17%; 7/41) or pandemics specifically (24%; 10/41). In addition, 18% (7/39) of hospital pharmacies had a business continuity plan ready before the pandemic and the same number (18%; 7/39) created a plan once it had begun. However, of the hospital pharmacies that had a pandemic or disaster management plan before the COVID-19 crisis, 56% (9/16) did not have a business continuity plan. This percentage rose to 70% (16/23) among hospital pharmacies that did not have a pandemic or disaster management plan before the pandemic. The details of the business continuity plans are listed in Table 2.

**Table 2:** What did the Business continuity plan consists of? (multiple choice)

	Total n (%)*
All key functions and tasks were listed, with the names of the persons responsible	9 (22)
Tasks which could, in great part, be carried out at home (teleworking) were identified	9 (22)
Tasks which could be put on hold and whose usual staff could be moved to other roles were identified	8 (20)
All key functions and tasks had pre-identified replacements or deputies	7 (17)
Job functions at risk of severe contamination were identified (direct contact with other persons or clients, etc.)	7 (17)
Tasks which must imperatively be carried out within the pharmacy itself were identified	6 (15)
Some staff were trained for new roles and tasks before any staff shortages	5 (12)
Some staff were trained for new roles and tasks because of absences or illness among the usual persons responsible	2 (5)
Key posts were assigned dual leadership	2 (5)
Tasks which could be carried out by external companies or contractors were identified	2 (5)

* Of 41 answers.

Most hospital pharmacies (95%; 39/41) created a steering committee to manage the crisis. This was often composed of the head pharmacist (61%; 25/41), a member of the pharmaceutical logistics unit (34%; 14/41), a representative from outside the pharmacy (32%; 13/41), a representative of the hospital’s general crisis management team (27%; 11/41), a representative from the cantonal authorities (22%; 9/41) and a member of the clinical pharmacy unit (20%; 8/41). Among the hospital pharmacies with a disaster management plan, 53% (9/17) created new paper or electronic dashboards or another management tool (e.g. an Excel^®^ spreadsheet) especially for the COVID-19 crisis, 24% (4/17) used previously prepared dashboards and 24% (4/17) had no dashboards at all. Of the hospital pharmacies without prior standard operating procedures, 61% (14/23) created dashboards specifically to manage the crisis. The information panels on those dashboards are listed in Table 3. It is worth noting that only two hospital pharmacies (5%; 2/43) had an information panel about risk management.

**Table 3:** Which information was presented on dashboard information panels prepared for managing the COVID-19 crisis? (multiple choice)

	Total n (%)*
Overview of essential stock items requiring monitoring	21 (49)
Summary report of the hospital’s situation	16 (37)
Situation at the pharmacy	16 (37)
Situation overview	13 (30)
General problems affecting the pharmacy	10 (23)
Number of pharmacy employees	8 (19)
Forward/provisional planning	8 (19)
Problems faced by different units in the pharmacy	7 (16)
Important contacts	7 (16)
List of key functions	6 (14)
Journal of events	5 (12)
Emergency measures	4 (9)
Problem assessment (synthesis of problems encountered)	2 (5)
Pending issues	2 (5)
Risks management	2 (5)
Task completion status	2 (5)
Schedule of the next situation updates	2 (5)

* Of 43 answers.

### Human resources management

Hospital guidelines were the main source of human resources management advice during the COVID-19 outbreak (70%, 30/43). The main changes carried out in human resources management are summarised in Table 4. Role changes occurred in the following fields: manufacturing disinfectants (bottle collection, disinfectant production, and filling done by pharmacists and volunteers), administrative work for ICUs (done by pharmacy technicians), and pharmacy technicians’ tasks on wards and logisticians’ drug distribution tasks (9% or 4/43 hospital pharmacies reassigned pharmacists to these tasks).

**Table 4:** How were your human resources managed during the crisis? (multiple choice)

	Total n (%)*
New activities had to be performed to respond to the specific needs of the crisis	29 (67)
Activities had to be reorganised	27 (63)
Staff with functions that permitted it were asked to work from home	25 (58)
Essential activities were performed on-site or by teleworking (non-urgent activities ceased)	24 (56)
Staff at risk were moved to safer environments (either home or restricted to low-risk activities)	20 (47)
Normal activities continued during the crisis	20 (47)
Pharmacy telephone numbers were rerouted, and access to the pharmacy computer network was provided to pharmacists working from home	11 (26)
Extra staff were recruited from among volunteers	11 (26)
Others**	8 (19)
A dedicated pandemic response team was set up	8 (19)
Extra staff were recruited from among Switzerland’s civil defense personnel	7 (16)
Substitute or replacement staff were organised (especially in key functions)	7 (16)
Shift teams were kept together to avoid any mixing of staff	5 (12)
Sick leave was compensated	5 (12)
Extra staff were recruited from among Switzerland’s civilian service personnel	4 (9)
Extra staff were recruited from among former employees	3 (7)
Extra staff were recruited from among retired hospital pharmacy staff	2 (5)
No changes in practice	2 (5)
A business continuity plan was developed at the start of the crisis	1 (2)
Extra staff were recruited from the Swiss Armed Forces	0 (0)
Free telephone numbers were set up for maintaining contact with staff, clients and suppliers	0 (0)

* Of 43 answers

**e.g. extra staff from other hospitals, free parking provided by local authorities to avoid public transport use, staff presence minimised to reduce virus transmission risk, paid leave for pharmacy technicians because of closed inpatient units

### Drug management

**Fig. 1:**
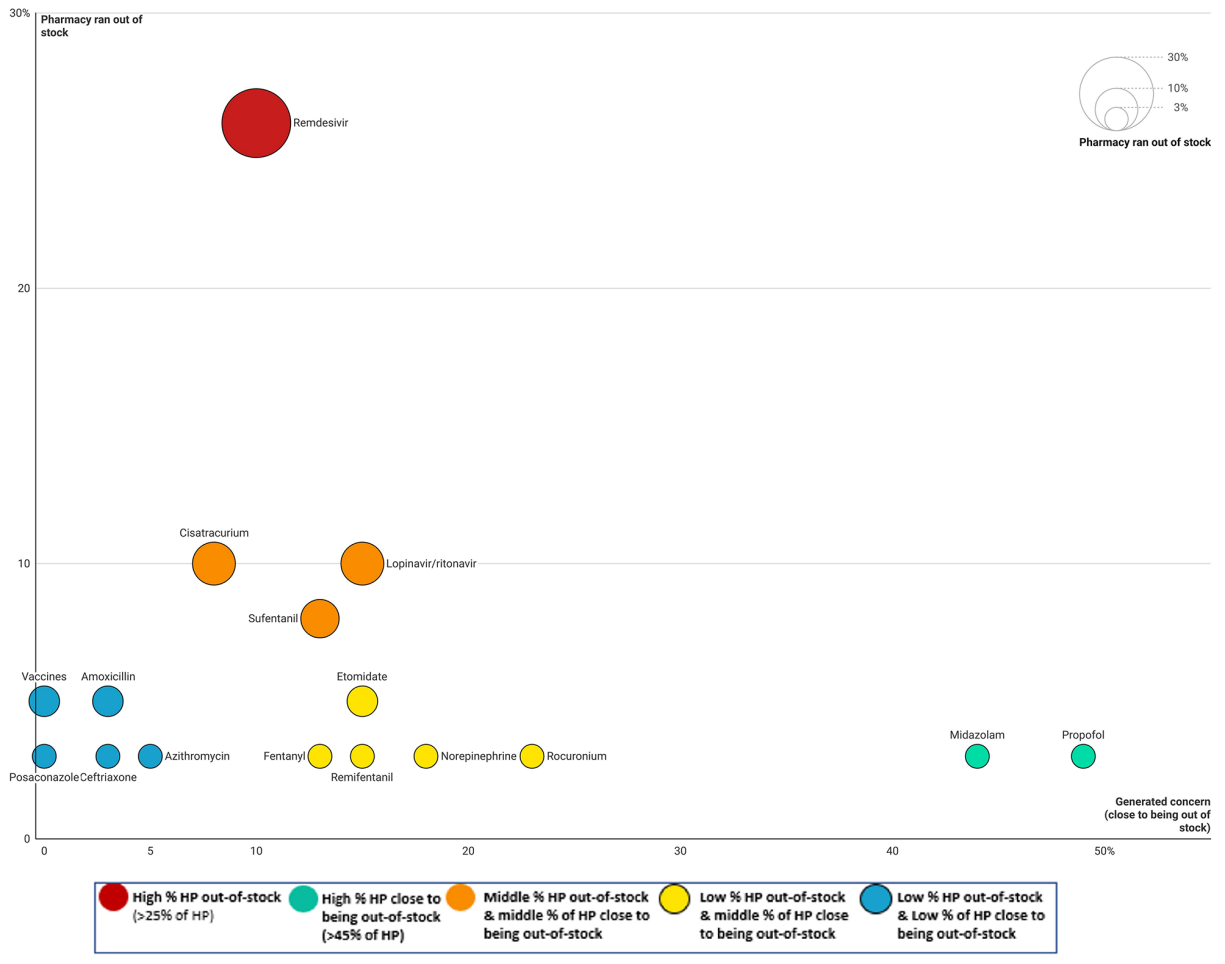
Drugs for the treatment of patients with COVID-19 that ran out of stock and/or generated concern

To limit the risks of drug shortages, 56% of hospital pharmacies (23/41) had reserve supplies. A list of drugs used for treating COVID-19 patients and their supply problems is shown in Fig. 1. Drugs were imported from the European Union by 34% of hospital pharmacies (14/41), but only 7% (3/41) had to import drugs from a country they did not usually deal with, and 10% (4/41) of hospital pharmacies asked a statistician to provide stock forecasts. Less than a quarter of hospital pharmacies (22%; 9/41) managed not to run out of any drug stocks at all. Drug stocks on wards treating COVID-19 patients were sometimes managed by assigning dedicated pharmacy technicians (29%; 12/41). Most hospital pharmacies (76%; 31/41) managed the risk of shortages by strictly monitoring the drugs dedicated to COVID-19 patients, while 63% (26/41) sourced alternative drugs and proposed these to medical staff or their healthcare institution instead. Alternatives were proposed to ensure the continuity of care and save stocks, with 27% of hospital pharmacies (11/41) having to prepare alternative protocols in partnership with medical or nursing care staff in case these potential shortages occurred. Half (51%; 20/39) of hospital pharmacies created specific drug lists for care units treating COVID-19 patients, and the types of drugs contained in these lists are summarised in Table 5.

Drug availability on COVID-19 wards was managed by increasing existing stocks (54% of pharmacies; 22/41) and creating extra storage space (51%; 21/41).

**Table 5:** Drugs used for treating COVID-19 patients in 2020

Therapeutic class	n (%)	Drugs
COVID-19 treatments	22 (83)	Lopinavir/ritonavir (and equivalents), hydroxychloroquine (and equivalents), remdesivir (tocilizumab)
Sedatives	21 (81)	Dexmedetomidine, lorazepam, midazolam
Anaesthetics	20 (77)	Etomidate, fentanyl, ketamine, propofol, remifentanil, sufentanil, suxamethonium
Antibiotics	19 (73)	Amikacin, amoxicillin, azithromycin, ceftriaxone, cefuroxime, imipenem/cilastatin, meropenem, piperacillin/tazobactam, posaconazole
Curares	15 (58)	Atracurium, cisatracurium, rocuronium
Electrolytes	11 (42)	ND
Perfusions	10 (38)	Heparin, insulin
Laxatives	4 (15)	ND
Others	3 (12)	Anticoagulation drugs, G-CSF and nutritional supplements

ND: No data

Drug production units in 24% of hospital pharmacies (10/41) reconditioned drugs that had not previously been done locally, and 10% (4/41) had to manufacture drugs, the main ones being all parenteral forms of hydromorphone, midazolam, morphine, ketamine and fentanyl plus hydroxychloroquine suspension.

### Support for medical and nursing care teams

Less than half (49%; 19/39) of hospital pharmacies worked with other clinical teams in their hospital to provide appropriate treatment management protocols for COVID-19 patients admitted to ICUs. Hospital pharmacies played a key role in many aspects of the COVID-19 pandemic by providing clinical and logistical support to medical and nursing care teams. Their detailed support activities for medical and nursing care teams are listed in Table 6.

**Table 6:** Support activities implemented by hospital pharmacies for medical and nursing care units

	Total n (%)*
Questions were dealt with by the usual pharmaceutical hotline	28 (68)
Specific documents were drawn up to respond to medical and nursing care staff’s needs regarding drug administration	12 (29)
Specific documents were drawn up to respond to medical and nursing care staff’s needs regarding drug preparation	12 (29)
A pharmacist was present in the ICU to support medical staff	10 (24)
Specific documents were drawn up to respond to medical and nursing care staff’s needs regarding treatment choices	10 (24)
A pharmacy technician was present in the ICU to help resupply wards with drugs	9 (22)
A pharmacist was present in the ICU to support care staff	8 (20)
A pharmacy technician was present in other care units treating COVID-19 patients to help resupply wards with drugs	8 (20)
A pharmacist was present in other care units treating COVID-19 patients to support medical staff	7 (17)
A pharmacist was present in other care units treating COVID-19 patients to support care staff	4 (10)
A pharmacy technician was present in other care units treating COVID-19 patients to help nurses withdraw drugs from their care unit’s drug stocks	2 (5)
A dedicated pharmacy hotline was set up to answer care staff’s questions linked to treating COVID-19	2 (5)
A pharmacy technician was present in the ICU to help nurses withdraw drugs from their care unit’s drug stocks	1 (2)
No specific support activities were proposed	7 (17)
A pharmacy technician was present in other care units treating COVID-19 patients to help nurses prepare drugs	1 (2)
A pharmacy technician was present in the ICU to help nurses prepare drugs (e.g. injectables)	0 (0)

* Of 41 answers

Overall, clinical pharmacy units also provided many tables with product characteristics and monitored every prescription of those specific drugs very closely (checking for interactions, contraindications and correct dosage).

### Other activities and problems reported on wards

About 20% of hospital pharmacies (8/41) implemented other support activities (stand-by services for ICUs and close communication with ICU heads). Pharmacy technicians took on some ICU administrative work or the centralised preparation of prefilled syringes for syringe pumps in support of nurses. Medication reviews for patient treatments were also carried out. One head hospital pharmacist sat on an interdisciplinary committee of physicians (emergency, ICU, pneumology, cardiology, infectious diseases and COVID-19 wards). Regarding problems involving the administration of drugs used mainly in ICUs, 12% of hospital pharmacies (5/41) experienced a lack of syringe pumps and 10% (4/41) experienced a lack of injectable drugs. Among the hospital pharmacies that faced problems involving the administration of drugs used mainly in ICUs, 60% (9/15) designed alternative drug administration protocols to save on the number of syringe pumps or injectable formulations used, 33% (5/15) managed the distribution and allocation of drug administration material, and 7% (1/15) developed protocols for administering remdesivir.

## Discussion

Most of Switzerland’s head hospital pharmacists answered our survey. Half of hospital pharmacies moved at-risk employees to safer environments, and a small number of participating hospital pharmacies set up a dedicated pandemic response team and provided pharmaceutical support to the medical and nursing teams on clinical wards. Some hospital pharmacies assigned a pharmacy team to perform daily medication stock control and management on clinical wards treating COVID-19 patients. To limit the risks of shortages in hospitals’ central pharmacies, just over half had planned reserve supplies.

Reorganising human resources was an important means of ensuring the continuity of pharmacy activities during the pandemic. In some countries [21], hospital pharmacies reorganised their activities to limit risks to their human resources: roles were redistributed and non-pharmacist staff were used to assist in manufacturing units and answer the phone [22]. Other hospital pharmacies encouraged working from home and physical distancing [23]. Personal fears regarding the risks of contamination originating from nursing homes or between colleagues were issues in some hospital pharmacies and should be considered in any future pandemic preparedness plans.

The presence of a team leader is crucial during crisis management [24, 25]. The Swiss Federal Office of Public Health’s pandemic preparedness plan recommends that organisations create a ‘pandemic team’ to manage their response to an outbreak [26]. However, our study revealed that most pandemic teams were composed solely of the head pharmacist and a representative of the pharmaceutical logistics unit (usually a pharmacist charged with hospital pharmacy logistics). One recent hospital pharmacy study highlighted the difficulties head pharmacists experienced when trying to lead crisis management. The leader and their team must quickly identify the problem and then make decisions, implement management tools and communicate effectively. Dashboards can be helpful for this [27]. Among our survey’s hospital pharmacies with a disaster management plan, half created paper or electronic dashboards or other management tools (e.g. an Excel^®^ spreadsheet) especially for the COVID-19 crisis. In contrast, few hospital pharmacies had prepared a human resources continuity plan. The recent study mentioned above also underlined the importance of preparing and performing drills. Although having a management plan is a precious starting point, it is not enough on its own [27]. The important thing is testing it and training with it [28].

Higher incidences of running out of stock were to be expected during a pandemic; thus, drug stocks in care units treating COVID-19 patients were managed by either increasing existing stocks or creating extra storage space. Drug management on wards treating COVID-19 patients forced some adjustments to routine roles. At the beginning of the pandemic, it was very difficult for hospital pharmacies to anticipate which drugs would be used the most and, thus, which had to be ordered, bought and stocked in sufficient quantities.

A scoping review of pharmacists’ roles during the pandemic suggested that hospital pharmacies provided drug information to other healthcare professionals as part of their daily activities [29]. This significant review was the first to highlight that counselling other healthcare professionals and patients on drugs was the main job performed by hospital pharmacists during the COVID-19 pandemic. Indeed, the outbreak revealed new opportunities for hospital pharmacists as they stepped into new roles (greater involvement in reflections about treatment choices and advising on strategies to limit drug consumption). This suggested that fully integrated, inter-sectoral, inter-professional collaboration is necessary to effectively face crises and public health emergencies [30]. Our study highlighted the key role played by hospital pharmacies across many aspects of the COVID-19 pandemic as they provided clinical and logistical support to medical and nursing care teams. Clinical and hospital pharmacists promoted safe, effective medication management for COVID-19 patients and participated in guideline development in partnership with other healthcare professionals (physicians, infectiologists, and other specialists) and pandemic management experts. Our study found that hospital pharmacists were physically present on clinical wards to support nurses and physicians. Their roles there were varied and included safeguarding sufficient stocks and proposing alternatives to ensure the continuity of care. Specific COVID-19 documents were also drawn up to respond to medical needs regarding drug administration, drug preparation and treatment choices. Indeed, by working together with physicians to provide appropriate treatment management protocols for COVID-19 patients admitted to ICUs, pharmacists cemented their important role during a pandemic. The importance of their roles is supported by the international disaster health community, which states that pharmacists should have a broader role than logistics in disaster response, including stockpile management, vaccinations, health and medication education, and ensuring continuity in medication care [7, 9].

Future improvements to Switzerland’s ability to respond to crises will require action at many levels: in hospital pharmacies, hospitals, cantons and nationally. To prevent running out of stock, the manufacture of drugs and medical devices within the country should be promoted, as should other technical and political measures. Overall, should there be any more waves of pandemic across Switzerland, be it COVID-19 or another pathogen, hospital pharmacists are recommended to think about the following measures today: (1) update standard operating procedures and emergency planning procedures; (2) develop worst-case scenario plans for dealing with supply chain problems and train for them (e.g. table-top preparedness exercises); (3) refine business continuity plans; (4) maintain the dashboards and concepts used in the first wave (e.g. communication concepts); (5) ensure that protection measures and hygiene and social distancing rules are respected; (6) anticipate needs and ensure minimal pharmacy stocks by preparing ‘pandemic inventories’ of drugs, disinfectants and personal protective equipment; (7) monitor and evaluate drug use, availability and shortages; and (8) talk directly with other stakeholders (wards, public administrations and so on).

The present survey had some limitations. The first was that only two-thirds of Switzerland’s head hospital pharmacists responded to the questionnaire despite two reminders. Nevertheless, this rate remains high and can be considered representative of pharmacies working in acute hospitals. Pharmacists who were not affiliated with the GSASA were not requested to participate because, as they generally do not work in acute care hospitals (but rather in psychiatric clinics or small private hospitals), they were potentially less affected by the imperatives of managing the COVID-19 crisis. Also, some participants failed to complete the whole survey, making comparisons between some answers difficult in a few cases.

## Conclusions

The present survey highlighted the key roles played by hospital pharmacies in many aspects of dealing with the pandemic, especially by providing major logistical and clinical support to physicians and nursing care teams. Hospital pharmacies generally found solutions quickly, effectively and safely in order to respond to this crisis and ensure the continuity of essential activities. Overall, our findings illustrated the wide range of activities undertaken by hospital pharmacies regarding crisis management (e.g. creating a pandemic management team, using standard operating procedures, showing flexibility and adaptability in reorganizing activities and teams), but they identified major areas of concern that emerged due to this public health crisis. The lessons and experiences outlined here could be used to improve the quality of pharmacies’ preparations for similar future events in Switzerland, Europe and worldwide.

### Electronic supplementary material

Below is the link to the electronic supplementary material.

Supplementary Material 1

Supplementary Material 2

## Data Availability

All the survey questions are available in Appendix I. Detailed results for each survey question are available from the corresponding author upon reasonable request.

## Abbreviations

COVID-19: Coronavirus disease 2019
EpidA: Swiss Federal Act on Epidemics
GSASA: Swiss Association of Public Health Administration and Hospital Pharmacists
ICU: Intensive care unit
SARS-CoV-2: Severe acute respiratory syndrome coronavirus 2
WHO: World Health Organization
