# Supplementary material for: Managing the COVID-19 health crisis: a survey of Swiss hospital pharmacies
Source: BMC Health Serv Res. 2023 Oct 20;23:1134. doi: 10.1186/s12913-023-10105-6 (PMC10589985; doi:10.1186/s12913-023-10105-6)
Supplement: Supplementary file 1 — Supplementary Material 1 [file 12913_2023_10105_MOESM1_ESM.pdf]

Introduction

Multicentric, prospective, observational study with the aim of evaluating how hospital pharmacies managed the crisis linked to the COVID-19 pandemic in Switzerland.

Management of the COVID-19 health crisis by hospital pharmacies in Switzerland

General Information

1. What are your family name, first name, e-mail address and hospital pharmacy name (optional) ?

Family Name :

First Name :

E-mail address :

Name of your hospital pharmacy :

* 2. Would you be willing to let us contact you should we require any additional details?

- Yes
- No

* 3. Which language region is your hospital pharmacy located?

- German
- French
- Italian
- Romansh

* 4. How many staff work in your hospital pharmacy?

- < 10
- 10-50
- 50-100
- >100

* 5. How many sites supply your pharmacy?

- 1
- 2-5
- 6-10
- >11

* 6. How many beds are there in your hospital(s) ?

	General Medicine	Intensive Care	Intermediate Care	Total
Standard arrangement				
Number of beds allotted to the management of the COVID-19 health crisis				

7. Please add any relevant details about your hospitals' structure here.

Management of the COVID-19 health crisis by hospital pharmacies in Switzerland

Management

* 8. Does your hospital pharmacy have a crisis management plan ?

- Yes, SOP
- Yes, pandemic plan
- No, We base ourselves on the Swiss Confederation’s plan (Pandemic: Preparedness handbook for enterprises)
- No

* 9. Did your hospital pharmacy trigger one of these plans?

- Yes
- No
- Partially

* 10. Which members of staff made up the steering committee/the management team/the crisis management unit?

- [ ] A representative of the drug production unit
- [ ] A representative of the clinical pharmacy unit and/or the pharmaceutical assistance unit
- [ ] A representative of the pharmaceutical logistics unit
- [ ] A crisis management assistant
- [ ] The head of the COVID-19 response project team
- [ ] The head pharmacist
- [ ] A representative of the hospital’s general crisis management team
- [ ] A representative of a higher authority (the hospital, the cantonal or federal level, etc.)
- [ ] A representative of a supplier
- [ ] A representative of the central warehouse
- [ ] A representative of the sterilisation unit
- [ ] Deputy head pharmacist
- [ ] Other representatives from outside of the pharmacy
- [ ] No steering committee/mangement team/crisis management

* 11. Did you use information panels/boards to help you manage this crisis? (i.e.: a set of synthetic paper or computerized documents on the general state of situation and indicators on the operation of the pharmacy)

- ○ Yes. We used panels/boards which had been prepared previously as a part of our internal crisis management plan
- ○ Yes. We used panels/boards which had been prepared previously as part of our internal crisis management plan, and we completed them with extra data
- ○ Yes. We created some panels/boards specially for this crisis
- ○ No

Management of the COVID-19 health crisis by hospital pharmacies in Switzerland

Management - *If panels/boards/documents were used*

12. Which reference works or guidelines did you use to help prepare panels/boards/documents ? (please specify)

* 13. Which information panels/boards/documents did you have available?

- [ ] Situation overview
- [ ] Summary hospital situation report
- [ ] Situation at the pharmacy
- [ ] General problems affecting the pharmacy
- [ ] Problems faced by different sectors, units or departments in the pharmacy
- [ ] Problems encountered (give details for each problem)
- [ ] Journal of events
- [ ] Number of pharmacy staff
- [ ] Emergency measures
- [ ] Outstanding issues
- [ ] Risks
- [ ] Provisional planning
- [ ] Task completion status
- [ ] Overview of essential stock items requiring monitoring
- [ ] Important contacts
- [ ] List of key functions
- [ ] Panel summarising the times of situation updates
- [ ] Others (please give details)

* 14. How would you evaluate usefulness of yours boards/panels/documents in relation to the following domains? (on a scale from 1–5, with 5 being the best)

	1	2	3	4	5
Well adapted to the situation	○	○	○	○	○
Gives a good general overview of the situation	○	○	○	○	○
Structures the information and challenges well	○	○	○	○	○
Structures the problems well	○	○	○	○	○

Management of the COVID-19 health crisis by hospital pharmacies in Switzerland

Management

* 15. Has your head pharmacist or a member of your company's management team been involved in a steering group at any of the following levels?

- [ ] Hospital
- [ ] Cantonal
- [ ] Federal
- [ ] None
- [ ] Other (please give details)

* 16. In general terms, how would you evaluate usefulness of the support of the authorities/affiliated agencies in the management of the health crisis at your level? (on a scale from 1–5, with 5 being the best)

	1	2	3	4	5	Not involved
The Federal Office of National Economic Supply (FONES)	○	○	○	○	○	○
The Federal Office of Public Health (FOHP)	○	○	○	○	○	○
Coordinated Medical Services/Medical Services Coordinating Body (CMS/SANKO)	○	○	○	○	○	○
Swissmedic	○	○	○	○	○	○
The cantonal public healthcare authorities as a whole	○	○	○	○	○	○
The cantonal pharmacist	○	○	○	○	○	○
The Swiss Association of Public Health Administration and Hospital Pharmacists (GSASA)	○	○	○	○	○	○
PharmaSuisse	○	○	○	○	○	○

17. Please use this box if you wish to give any more details about your hospital pharmacy's crisis management plan.

Human Resources

* 18. Have any of your staff had to take sick leave because they were infected by COVID-19?

- ◯ None
- ◯ < 5% of the workforce
- ◯ 6–10% of the workforce
- ◯ 11–20% of the workforce
- ◯ > 20% of the workforce

19. What basis did you use to manage your human resources?

- ☐ Your hospital's guidelines
- ☐ Your hospital pharmacy's internal guidelines
- ☐ Other (please give details)

* 20. How have you managed your human resources during the crisis?

- ☐ A dedicated pandemic response team was set up
- ☐ People at risk were moved to safer environments (either home or restricted to low-risk activities)
- ☐ Staff members with functions which permitted it were asked to work from home
- ☐ Pharmacy telephone numbers were rerouted, and access to the pharmacy computer network was provided
- ☐ Extra human resources were recruited from among retired personnel
- ☐ Extra human resources were recruited from among former employees
- ☐ Extra human resources were recruited from among volunteers
- ☐ Extra human resources were recruited from among civil protection personnel
- ☐ Extra human resources were recruited from the army
- ☐ Extra human resources were recruited from among civilian service personnel
- ☐ Activities had to be reorganised
- ☐ Only essential activities were maintained (non-urgent activities were shut down)
- ☐ Regular activities were able to be maintained during the crisis
- ☐ New activities had to be put in place to respond to the specific needs created by the crisis
- ☐ Shift teams were kept together to avoid any mixing of personnel
- ☐ Substitute or replacement pf staff members were organised (especially in key functions)
- ☐ Sick leave compensation
- ☐ Free telephone numbers were set up for maintaining contact with staff, clients and suppliers
- ☐ No change in practice
- ☐ Others (please specify)

* 21. What percentage (on average) of your workforce was attributed to the following activities at the peak of the infection?

	Routine activities	COVID-19 activities
Pharmacists		
Pharmacy assistants		
Pharmacy assistants on wards or in care units		
Logisticians		
Quality controllers		

* 22. What percentage (on average) of your workforce had to change jobs at the peak of the infection? For example, pharmacists who had to do the work of pharmacy assistants.

- 0%
- 1–10%
- 11–20%
- 21–30%
- 31–50%
- > 50%

23. What job changes occurred? (please give details)

* 24. Did you have to put in place psychological support measures for your teams?

- Measures were proposed by our overarching management structure (the hospital, the canton, etc.)
- A telephone helpline was set up
- More flexible work scheduling was put in place
- Specific hygiene measures were put in place
- Team briefings and debriefings were introduced
- Staff were able to request a personal consultation if they felt the need
- Situation updates were given regularly and their consequences explained to teams in order to stop rumours
- The personnel were calmed and reassured, and a climate of trust was established
- Solidarity between the personnel was promoted
- No specific measures
- Others (please specify)

* 25. How did you manage your staffs' holidays, leave and days off?

- [ ] Our institution cancelled holidays, leave and days off for a certain time
- [ ] Some short periods of leave were accorded in order to give staff some relief throughout the crisis
- [ ] Most of the holidays requested by staff were granted
- [ ] During the crisis, staff were obliged to use up all of their leave and accumulated overtime hours as initially planned
- [ ] Others (please specify)

* 26. Do you have a business/activity continuity plan?

- ( ) Yes. The plan existed before the pandemic occurred
- ( ) Yes. The plan was specially created to manage the pandemic
- ( ) No

Management of the COVID-19 health crisis by hospital pharmacies in Switzerland

Human Resources - *If your pharmacy does have a buisiness/activity continuity plan*

* 27. What did it consist of ?

- [ ] All the key functions and tasks were listed with the names of the persons responsible
- [ ] All the key functions and tasks had pre-identified replacements or deputies
- [ ] Some staff members were trained for new functions and tasks before any staff shortfalls
- [ ] Some staff members were trained for new functions and tasks because of absences or illness among the usual persons responsible
- [ ] Key posts were assigned dual leads
- [ ] The tasks which must imperatively be carried out within the pharmacy itself were identified
- [ ] The tasks which could, in great part, be carried out at home (teleworking) were identified
- [ ] The tasks which could temporarily be put on hold and whose usual workers could be moved to other roles were identified
- [ ] The tasks which could be carried out by external companies or contractors were identified
- [ ] The functions at risk of severe contamination were identified (direct contact with other persons or clients, etc.)
- [ ] Others (please specify)

Management of the COVID-19 health crisis by hospital pharmacies in Switzerland

Human Resources

28. Please use this box if you wish to give any more details about the human resources problems you faced.

Management of the COVID-19 health crisis by hospital pharmacies in Switzerland

Drugs mainly used in Intensive Care Units

* 29. Do you have specific treatment management protocols for patients with COVID-19 arriving in your intensive care unit?

- Yes. The hospital provided us with protocols without consulting us
- Yes. We worked together with our hospital to put in place appropriate protocols
- Yes. We have a protocol, but it's from an other source
- No. There was no specific protocol available

* 30. There may have been some procurement or supply problems during this time. Which drugs were most affected by these in your hospital pharmacy?

	No problems were encountered with this drug	Closely monitored to avoid any risk of being out of stock	Generated concern (close to being out of stock)	Pharmacy ran out of stock
Midazolam				
Propofol				
Fentanyl				
Rocuronium				
Atracurium				
Cisatracurium				
Remdesivir				
Ketamine				
Dexmedetomidine				
Etomidate				
Sufentanil				
Remifentanil				
Suxamethonium				
Norepinephrine				
Adrenaline				
Insulin				
Fentanyl				

	No problems were encountered with this drug	Closely monitored to avoid any risk of being out of stock	Generated concern (close to being out of stock)	Pharmacy ran out of stock
Heparin	○	○	○	○
Morphine	○	○	○	○
Lorazepam	○	○	○	○
Amoxicillin	○	○	○	○
Piperacillin/Tazobactam	○	○	○	○
Meropenem	○	○	○	○
Imipenem/Cilastatin	○	○	○	○
Cefuroxime	○	○	○	○
Ceftriaxone	○	○	○	○
Amikacin	○	○	○	○
Posaconazole	○	○	○	○
Vaccines	○	○	○	○
Medical gases	○	○	○	○
Other	○	○	○	○

If others (please specify the name of drugs)

* 31. How did you manage being out of stock?

- [ ] A specific team was set up
- [ ] Extra resources were allotted to this problem
- [ ] Alternative drugs were found and proposed to medical personnel or the healthcare institution
- [ ] My institution prepared alternative protocols to make up for these shortages, in partnership with medical or care personnel
- [ ] Medical personnel imposed their choices of compounds and/or their corresponding protocols
- [ ] Reserve supplies had been anticipated to limit the risks of any shortages
- [ ] Drugs were made by the drug production unit
- [ ] Drugs were repackaged by the drug production unit
- [ ] These drugs were strictly monitored daily or weekly
- [ ] A statistician was asked to help provide stock forecasts
- [ ] Drugs were imported from a European Union country
- [ ] Drugs were imported from another authorised country
- [ ] Drugs were imported from a country outside those usually authorised
- [ ] No drugs out of stock
- [ ] Others (please specify)

32. If you had to manufacture drug s in your pharmacy on the basis of the raw material, which drug was it?

* 33. Please rate how you feel about the support received from the following competent authorities/affiliated agencies with regards to the management of shortages of drugs mainly used in Intensive Care Units? (on a scale from 1–5, with 5 being the best)

	1	2	3	4	5	Not involved
The Federal Office of National Economic Supply (FONES)	○	○	○	○	○	○
The Federal Office of Public Health (FOHP)	○	○	○	○	○	○
Coordinated Medical Services/Medical Services Coordinating Body (CMS/SANKO)	○	○	○	○	○	○
The Swiss Association of Public Health Administration and Hospital Pharmacists (GSASA)	○	○	○	○	○	○
The cantonal pharmacist	○	○	○	○	○	○
The cantonal public healthcare authorities as a whole	○	○	○	○	○	○
Swissmedic	○	○	○	○	○	○
PharmaSuisse	○	○	○	○	○	○

* 34. Did you have any problems involving the administration of drugs mainly used in Intensive Care Units?

- ☐ Yes. There was a lack of syringe pumps or syringes, etc.
- ☐ Yes. There was a lack of injectable drugs
- ☐ Yes. Care teams lacked experience in dealing with these drugs
- ☐ Yes. Other reasons
- ☐ No

Management of the COVID-19 health crisis by hospital pharmacies in Switzerland

Drugs mainly used in Intensive Care Units - If you have been confronted with administration problem

35. How did you deal with this problem?

- ☐ We designed alternative drug administration protocols to save on the number of syringe pumps or injectable formulations used
- ☐ We managed the distribution and allocation of drug administration material
- ☐ Others reasons (please specify)

Drugs mainly used in Intensive Care Units

36. Please use this box if you wish to give any more details about the problems you faced concerning the drugs mainly used in Intensive Care Units.

Management of the COVID-19 health crisis by hospital pharmacies in Switzerland

Drugs used specifically for treating SARS-CoV-2

* 37. Did you use specific treatment management protocols for COVID-19 patients?

- Yes. The hospital provided us with protocols without consulting us
- Yes. We worked together with our hospital to put in place appropriate protocols
- Yes. We have a protocol, but it's from an other source
- No. There was no specific protocol available

* 38. Did you experience any problems procuring drugs used for treating specifically Covid-19 patients?

	No problems were encountered with this drug	Closely monitored to avoid any risk of being out of stock	Generated concern (close to being out of stock)	Pharmacy ran out of stock
Kaletra® (lopinavir/ritonavir) and equivalents				
Plaquenil® (hydroxychloroquine) and equivalents				
Tocilizumab (Actemra®)				
Remdesivir				
Azithromycin				
Others				

If others (please specify the name of drugs)

* 39. How did you manage being out of stock?

- [ ] A specific team was set up
- [ ] Extra resources were allotted to this problem
- [ ] Alternative drugs were found and proposed to medical personnel or the healthcare institution
- [ ] My institution prepared alternative protocols to make up for these shortages, in partnership with medical or care personnel
- [ ] Medical personnel imposed their choices of compounds and/or their corresponding protocols
- [ ] Reserve supplies had been anticipated to limit the risks of any shortages
- [ ] Drugs were made by the drug production unit
- [ ] Drugs were repackaged by the drug production unit
- [ ] These drugs were strictly monitored daily or weekly
- [ ] A statistician was asked to help provide stock forecasts
- [ ] Drugs were imported from a European Union country
- [ ] Drugs were imported from another authorised country
- [ ] Drugs were imported from a country outside those usually authorised
- [ ] No drugs out of stock
- [ ] Others (please specify)

40. If you had to manufacture drug s in your pharmacy on the basis of the raw material, which drug was it?

* 41. Please rate how you feel about the support received from the following competent authorities/affiliated agencies with regards to the management of shortages of drugs used for the treatment of SARS-CoV-2? (on a scale from 1–5, with 5 being the best)

	1	2	3	4	5	Not involved
The Federal Office of National Economic Supply (FONES)	○	○	○	○	○	○
The Federal Office of Public Health (FOHP)	○	○	○	○	○	○
Coordinated Medical Services/Medical Services Coordinating Body (CMS/SANKO)	○	○	○	○	○	○
The Swiss Association of Public Health Administration and Hospital Pharmacists (GSASA)	○	○	○	○	○	○
The cantonal pharmacist	○	○	○	○	○	○
The cantonal public healthcare authorities as a whole	○	○	○	○	○	○
Swissmedic	○	○	○	○	○	○
PharmaSuisse	○	○	○	○	○	○

* 42. Were you involved in either the Solidarity or Discovery studies or another clinical study?

- ○ Yes
- ○ No

Management of the COVID-19 health crisis by hospital pharmacies in Switzerland

Drugs used specifically for treating SARS-CoV-2 - *If you have been involved in a clinical study*

* 43. What project/study did you participate ?

- ☐ Solidarity
- ☐ Discovery
- ☐ Early Access Program Remdesivir
- ☐ #StayHome
- ☐ Others (please specify)

* 44. How were you involved?

- [ ] Our hospital participated in the study/project
- [ ] We had to provide the drugs for the study/project
- [ ] We had a pharmacist consulting for the study/project
- [ ] We worked on this project in partnership with medical and care personnel

Management of the COVID-19 health crisis by hospital pharmacies in Switzerland

Drugs used specifically for treating SARS-CoV-2

45. Please use this box if you wish to give any more details about the problems you faced concerning drugs used specifically for the treatment of the SARS-CoV-2 virus?

Management of the COVID-19 health crisis by hospital pharmacies in Switzerland

Drug management in COVID-19 care units (including Intensive Care Units)

* 46. Did you create specific drug lists for care units treating COVID-19 patients?

- ( ) Yes
- ( ) No
- ( ) Partly

Management of the COVID-19 health crisis by hospital pharmacies in Switzerland

Drug management in COVID-19 care units - *If you have been created specific drug lists*

47. Which types of drugs did they contain?

- [ ] Sedatives
- [ ] Curare-type drugs
- [ ] Anaesthetics
- [ ] Antibiotics
- [ ] Laxatives
- [ ] Electrolytes
- [ ] Perfusions
- [ ] "COVID-19" treatments such as hydroxychloroquine, lopinavir/ritonavir or others
- [ ] Others (please specify)

Management of the COVID-19 health crisis by hospital pharmacies in Switzerland

Drug management in COVID-19 care units (including Intensive Care Units)

* 48. How did you manage drug stocks in care units treating COVID-19 patients?

- [ ] We created extra storage space
- [ ] We increased existing stocks
- [ ] Others (please specify)

* 49. How did you manage drug stocks in care units treating COVID-19 patients with regards to human resources?

- [ ] We assigned dedicated pharmacy assistants to those care units
- [ ] We hired extra human resources for those tasks
- [ ] We reassigned some pharmacy assistants so that they split their time between COVID-19 care units and their usual tasks
- [ ] We reassigned pharmacists to these tasks
- [ ] We reassigned these tasks to other professionals (nurses or others)
- [ ] Others (please specify)

50. Please use this box to provide a detailed description of how you organised drug management in care units treating COVID-19 patients

51. Please use this box if you wish to give any more details about the problems you faced concerning the drugs used in care units treating COVID-19 patients.

Management of the COVID-19 health crisis by hospital pharmacies in Switzerland

Hygiene

* 52. Did you experience any problems procuring hand disinfection solution?

- ◯ No. We do not manage this type of product
- ◯ No. We had no problems procuring this type of product
- ◯ Yes

Management of the COVID-19 health crisis by hospital pharmacies in Switzerland

Hygiene - *If you have been exeperiencing any problems with hand disinfection solution*

* 53. What were your most significant problems?

	Not significant	Significant	Very significant
Obtaining basic ingredients	◯	◯	◯
Labour	◯	◯	◯
Empty bottles	◯	◯	◯
Infrastructure	◯	◯	◯
Distribution	◯	◯	◯
Theft	◯	◯	◯
Over-consumption	◯	◯	◯
Others	◯	◯	◯

If others (please specify the name of drugs)

* 54. How did you manage these problems?

- ☐ We manufactured it ourselves
- ☐ We imported it
- ☐ We outsourced work to external companies
- ☐ We received donations from outside the institution
- ☐ Others (please specify)

Management of the COVID-19 health crisis by hospital pharmacies in Switzerland

Hygiene - Protective masks

* 55. Were you involved in the management and distribution of protective masks?

- ○ Yes
- ○ No
- ○ Partly

56. What was your involvement?

- ☐ Distribution of state stocks at the cantonal or regional level
- ☐ Providing information to healthcare professionals
- ☐ Buying and distribution of private stocks
- ☐ Manufacturing
- ☐ Others (please specify)

Management of the COVID-19 health crisis by hospital pharmacies in Switzerland

Hygiene

* 57. Were you involved in issues concerning surface disinfectants?

- ☐ No. We do not manage this type of product
- ☐ Partly. We were consulted for our expert opinion
- ☐ Yes. We usually manage stocks of this type of product
- ☐ Yes. We manufactured surface disinfection solutions
- ☐ Yes. We prepared documentation with guidelines on selecting disinfectants
- ☐ Yes. We managed overall stocks of this type of product (at the national or cantonal level)
- ☐ Others (please specify)

* 58. Did you contribute to setting up internal procedures in your institution concerning personal protective equipment (PPE)?

- ○ Yes. At our own initiative
- ○ Yes. We followed the FOPH recommendations, our hospital plan or the Swiss Confederation’s plan (Pandemic: Preparedness handbook for enterprises)
- ○ No

* 59. Did you contribute to setting up internal procedures in your hospital pharmacy concerning workplace hygiene (the disinfection of surfaces and equipment such as computers)?

- ○ Yes. At our own initiative
- ○ Yes. Based on the hospital's guidelines
- ○ Yes. We followed the FOPH recommendations, our hospital plan or the Swiss Confederation’s plan (Pandemic: Preparedness handbook for enterprises)
- ○ No

60. Please use this box if you wish to give any more details about the problems you encountered with regards to hygiene.

[ ]

Management of the COVID-19 health crisis by hospital pharmacies in Switzerland

Support for medical and care teams

* 61. Did you implement any support activities for specific care units?

- ☐ A dedicated pharmacy hotline was set up to answer care staff’s questions linked to treating COVID-19
- ☐ Questions were dealt with our usual pharmaceutical hotline
- ☐ A pharmacist was present in the ICU to support medical personnel
- ☐ A pharmacist was present in the ICU to support nurses
- ☐ A pharmacy assistant was present in the ICU to help nurses withdraw drugs from their care unit drug stocks
- ☐ A pharmacy assistant was present in the ICU to help nurses prepare drugs (e.g. preparation of injectables, preparation of weekly pill-boxes, etc.)
- ☐ A pharmacy assistant was present in the ICU to help resupply wards with drugs
- ☐ A pharmacist was present in other care units treating COVID-19 patients to support medical personnel
- ☐ A pharmacist was present in other care units treating COVID-19 patients to support nurses
- ☐ A pharmacy assistant was present in other care units treating COVID-19 patients to help nurses withdraw drugs from their care units drug stocks
- ☐ A pharmacy assistant was present in other care units treating COVID-19 patients to help nurses prepare drugs
- ☐ A pharmacy assistant was present in other care units treating COVID-19 patients to help resupply wards with drugs
- ☐ Specific documents were drawn up to respond to medical and care personnel’s needs with regards to drug administration
- ☐ Specific documents were drawn up to respond to medical and care personnel’s needs with regards to treatment choices
- ☐ Specific documents were drawn up to respond to medical and care personnel’s needs with regards to drug preparation
- ☐ No specific support activities were proposed
- ☐ Other support activities were put in place (please specify)

[ ]

62. Please use this box if you wish to give any more details about the support you gave to care units, the requests which you received or the problems which you encountered.

[ ]

Management of the COVID-19 health crisis by hospital pharmacies in Switzerland

Care management for patients recovering from COVID-19

* 63. Did your institution prepare specific treatment management plans for patients recovering from COVID-19?

- [ ] Implementation of specific monitoring for long-term (> 3 weeks in an ICU) COVID-19 patients
- [ ] An extra intermediate care unit was opened
- [ ] An extra rehabilitation unit was opened
- [ ] Implementation of specific post-COVID-19 cardiological monitoring
- [ ] Implementation of specific post-COVID-19 neurological monitoring
- [ ] Others (please specify)
- [ ] Implementation of specific out-patient monitoring for patients discharged home (Covicall, Covicheck, Covicare)
- [ ] Implementation of specific telemedicine solutions for patients discharged home
- [ ] I don’t know
- [ ] None

Management of the COVID-19 health crisis by hospital pharmacies in Switzerland

Other problems encountered

64. Did you encounter any other notable problems not covered in any of the preceding questions?

Management of the COVID-19 health crisis by hospital pharmacies in Switzerland

Future perspectives

65. What have you done to prepare for a potential second wave of infections?

66. What do you think should be done to improve future crisis response:

In your hospital pharmacy?	
In your hospital?	
At the cantonal level?	
At the Swiss federal level?	
Others level	

67. What would you expect from the Specialised Centre for Emergency and Disaster Pharmacy (http://www.disaster-pharmacy.ch/) ?
