# Supplementary material for: Managing the COVID-19 health crisis: a survey of Swiss hospital pharmacies
Source: BMC Health Serv Res. 2023 Oct 20;23:1134. doi: 10.1186/s12913-023-10105-6 (PMC10589985; doi:10.1186/s12913-023-10105-6)
Supplement: Supplementary file 2 — Supplementary Material 2 [file 12913_2023_10105_MOESM2_ESM.pdf]

Specific results for Switzerland

Table 7 Main results and differences between Switzerland's German-speaking (G) and French- and Italian-speaking (F/I) regions

		German n (%)	French/Italian n (%)
**Crisis management plan (G n = 33; F/I n = 8)**	Yes, SOP	4 (13)	3 (37.5)
	Yes, Pandemic Plan	9 (27)	1 (12.5)
	No	20 (60)	4 (50)
**COVID-19 pandemic triggered crisis management plan, if it existed (G n = 33; F/I n = 8)**	Yes	5 (15)*	3 (38)*
	Partially	8 (24)*	4 (50)*
	No	20 (61)*	1 (13)*
**Business/activity continuity plan (G n = 31; F/I n = 8)**	Yes. Plan existed before pandemic	4 (13)*	3 (38)*
	Yes. Plan created to manage pandemic	4 (13)*	3 (38)*
	No	23 (74)*	2 (25)*
**Percentage (average) of workforce who had to change jobs at the peak of infection (G n = 33; F/I n = 8)**	0%	13 (42)	3 (38)
	1–20%	11 (36)	3 (38)
	20–50%	6 (20)	2 (26)
	> 50%	1 (3)	0 (0)
**Storage (G n = 33; F/I n = 8)**	Anticipated reserve supplies	15 (45)*	8 (100)*
	Imported drugs from the European Union	9 (27)*	5 (63)*
	Prepared alternatives protocols in case of shortages	7 (21)*	4 (50)*
**Pharmaceutical support of medical and care units (G n = 33; F/I n = 8)**	Pharmacy technician seconded to		
	ICU	6 (18)*	3 (38)*
	COVID-19 units	5 (15)*	3 (38)*
	Pharmacist support to		
	nursing care staff in ICU/ COVID-19 units	3 (9)*/ 1(3)*	5 (63)*/ 3(38)*
	medical staff in ICU/ COVID-19 units	4 (12)*/ 3 (9)*	6 (75)*/ 4(50)*
	Create documents regarding treatment choices	6 (18)*	4 (50)*

* Hospital pharmacies in French-speaking regions implemented their crisis management plans more frequently than those in German-speaking regions. This reflected the fact that French-speaking regions suffered more contaminations than German-speaking ones and thus hospital pharmacies there felt less need to create those plans.

Table 8 Level of satisfaction with national support (on a scale from 1–5, with 5 being the best)

		German (n = 31)	French/Italian (n = 8)
**Usefulness of the support received from national authorities or affiliated agencies in the management of the health crisis**	FONES	2.1	1.9
	FOHP	2.6	3.0
	CMS/SANKO	2.2	2.1
	Swissmedic	1.9	1.6
	Cantonal public healthcare	2.6	3.3
	Cantonal Pharmacist	3.6	3.8
	GSASA	4.2	4.4
	Pharmasuisse	2.4	1.6
**Support received from the competent national authorities or affiliated agencies for the management of shortages of drugs mainly used in intensive care units**	FONES	2.0	1.8
	FOHP	2.2	3.0
	CMS/SANKO	2.0	1.3

	Swissmedic	1.8	1.4
	Cantonal public healthcare	2.4	2.1
	Cantonal Pharmacist	3.4	4.0
	GSASA	3.9	3.8
	pharmaSuisse	1.6	1.0
**Support received from the competent national authorities or affiliated agencies for the management of shortages of drugs used for the treatment of SARS-CoV-2**	FONES	2.2	1.4
	FOHP	1.9	2.4
	CMS/SANKO	1.9	2.1
	Swissmedic	1.7	1.0
	Cantonal public healthcare	2.0	2.6
	Cantonal Pharmacist	3.0	3.4
	GSASA	3.7	3.0
	pharmaSuisse	1.3	0.6

FONES = Federal Office for National Economic Supply; **FOHP** = Federal Office of Public Health; **CMS/SANKO** = Coordinated Medical Service/Medical Services Coordinating Committee; **GSASA** = Swiss Association of Public Health Administration and Hospital Pharmacists

Table 9 Inputs to improve future crisis response in Switzerland at different levels

**Hospital pharmacy level**	**Hospital level**
➢ Better management of home office work<br>➢ Careful management of human resources<br>➢ Consider the risks of being out of stock when choosing medicines<br>➢ Establish/update a crisis management plan<br>➢ Establish/update a pandemic plan<br>➢ Identify key roles and tasks to facilitate reorganisation in the event of a crisis<br>➢ Re-evaluate definitions of “critical/essential” drugs and their stock level<br>➢ Improve communication on stock management	➢ Update/adapt pandemic plan<br>➢ Cooperate with physicians to deal with drug shortages<br>➢ Increase stocks of masks, gloves, disinfectant, PPE<br>➢ Involve the pharmacy in the crisis committee<br>➢ Designate clear leaders and define their key functions from the beginning<br>➢ Establish good communication
**Cantonal level**	**Swiss Federal level**
➢ Establish clearer, better communication between hospitals<br>➢ Define the responsibilities and funding of new requirements<br>➢ Establish and distribute protocols/SOPs for drug shortages<br>➢ Enable support by the Swiss Armed Forces for hospital pharmacies<br>➢ Establish a hotline for questions regarding the management and distribution of material and drugs<br>➢ Provide information and transparency on available drugs stocks<br>➢ Organise centralised stocks for crises in existing institutions (drugs, PPE, disinfectants)<br>➢ Interfere less in the routine management of drug procurement	➢ Be more reactive, anticipate better and communicate more clearly<br>➢ Constitute a strong central management committee with the involvement of hospital pharmacies at a decision-making level<br>➢ Coordinate and prepare from the start of the crisis<br>➢ Define the responsibilities and coordinate the activities of the federal offices of the armed forces of the cantons.<br>➢ Better involve pharmacists at different levels (hospital, GSASA, cantonal, army)<br>➢ Oblige pharmaceutical companies/suppliers to maintain larger stocks of essential medicines

SOP = Standard operating procedures; **PPE** = Personal protective equipment; **GSASA** = Swiss Association of Public Health Administration and Hospital Pharmacists
